# Mysterious Left Atrial Mass in a Patient With Metastatic Prostate Cancer

**DOI:** 10.7759/cureus.23238

**Published:** 2022-03-16

**Authors:** Rafsan Ahmed, Seyed Zaidi, Michelle Feinberg, Suzette Graham-Hill

**Affiliations:** 1 Internal Medicine, State University of New York (SUNY) Downstate Medical Center, Brooklyn, USA; 2 Cardiology, State University of New York (SUNY) Downstate Medical Center, Brooklyn, USA; 3 Neurosurgery, Kings County Hospital Center, Brooklyn, USA; 4 Cardiology, Kings County Hospital Center, Brooklyn, USA

**Keywords:** metastatic prostate cancer, myxoma, transesophageal echo, transthoracic echocardiogram, cardiac thrombi, left atrial mass

## Abstract

Cardiac tumors (CTs) are a rare group of disorders that encompass a broad set of masses. They are subclassified into neoplastic and non-neoplastic lesions. Neoplastic lesions can be further subdivided into either primary cardiac tumors (PCTs) or secondary cardiac tumors (SCTs) which are metastasis to the heart. Cardiac myxomas are the most common pathological type of benign PCT followed by rhabdomyomas, papillary fibroelastomas, fibromas, lipomas, and leiomyomas. Here, we present a case of a patient with left atrial mass in the setting of stage IV prostate cancer. We have used transthoracic echocardiography (TTE) and transesophageal echocardiography (TEE) for characterization and differential generation. Our findings are presented in high-quality imaging and video and our top differentials include PCT, thrombi, and metastasis. Although a full diagnostic workup was not completed due to limitations in diagnostic tests available, metastasis to the heart could not be excluded due to the high staging and extensive sclerotic involvement of this malignancy. We emphasized the importance of multimodality imaging, e.g., TTE, TEE, cardiac magnetic resonance (CMR), and cardiac computed tomography (CT) in the workup of incidental cardiac masses and differential refinement.

## Introduction

Although immensely rare, cardiac tumors encompass a broad set of masses, for which diagnostic and therapeutic management remains a challenge [1]. Cardiac tumors are subclassified into neoplastic and non-neoplastic lesions. Neoplastic lesions can be further subdivided into either primary or secondary tumors (i.e., metastatic involvement of the heart). Primary cardiac tumors (PCTs) can exist as either benign, malignant, or intermediate tumors. The incidence of PCTs is 1.38 new cases per 100,000 residents per year [2]. More than 90% of PCTs are benign, less than 10% are malignant and approximately 1% are intermediate in nature [3-5]. Secondary cardiac tumors are 20 times more common compared to PCTs [6]. In a study by Klatt and Heitz, metastasis to the heart was found in 10.7% of 1029 autopsy cases with diagnosed malignant neoplasm [7]. Malignant melanomas have the greatest inclination for cardiac metastasis, whereas lung, esophageal, and breast carcinomas are the most common carcinomas of the thorax to metastasize to the heart [7,8]. To date, there are no reports showing cardiac metastasis in the setting of prostate cancer. Here, we present a case of a patient with stage IV prostate cancer who was found to have an undiagnosed pathologic mass in the left atrium.

## Case presentation

An 88-year-old man with a history of hypertension, diabetes mellitus, and stage IV prostate cancer with extensive sclerotic metastasis was brought to the emergency department after being found lying on the kitchen floor. Upon arrival at the emergency department, mental status was notably altered from his baseline, blood pressure was 144/88, temperature was 93°F, respiratory rate was 32, and oxygen saturation was 88% which increased to 100% on room air. The patient had multiple forehead, facial, and bilateral lower extremity abrasions. A full neurological examination was limited due to the patient's inability to follow commands. His laboratory values are represented in Table 1. Initial troponin was 0.203 ng/mL initially which downtrend to 0.135 ng/mL. A CT scan of the head revealed acute intraparenchymal hemorrhages in the left midbrain, pons, and superior medulla, extending into the left inferior cerebellar peduncle and bilateral occipital intraventricular hemorrhages, as well as left phthisis bulbi. EKG was remarkable for atrial fibrillation with ST-T wave abnormalities in the lateral leads. The patient was admitted to the neurological intensive care unit for further care.

**Table 1:** Laboratory values of the patient on admission.

Laboratory test	Value on admission	Reference range
Sodium (mEq/L)	132	135-145
Potassium (mEq/L)	5.5	3.7-5.2
Blood urea nitrogen (mg/dL)	43	6-20
Creatinine (mg/dL)	2.2	0.6-1.3
Anion gap (mmol/l)	25	8-16
Alkaline phosphatase (U/L)	441	20-130
Aspartate aminotransferase (U/L)	99	8-33
Total bilirubin (mg/dL)	2.1	0.1-1.2
Total creatine kinase (U/L)	1509	22-198
Initial troponin (ng/mL)	0.203	0-0.04

**Figure 1:**
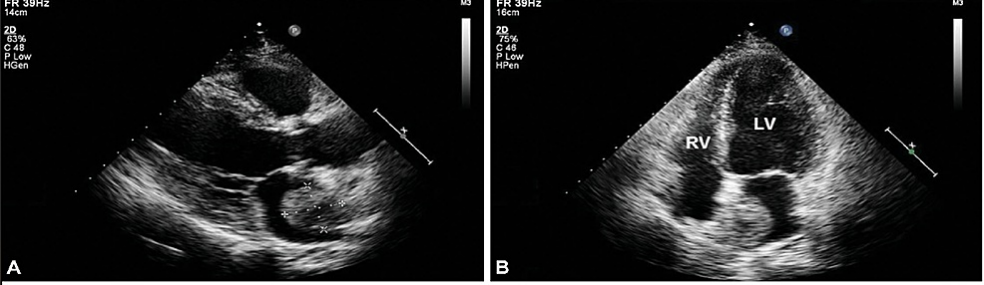
Parasternal long view (A) and apical four-chamber view (B) from the TTE. In both images, a severely dilated left atrium can be seen containing a large mobile mass attached to the wall. TTE: transthoracic echocardiography

Transthoracic echocardiography (TTE) performed showed mildly decreased left ventricular ejection fraction of 40-45%, mild left ventricular hypertrophy (Figure 1). Normal right ventricular systolic function and mild mitral, tricuspid, and pulmonic valve regurgitation. Pulmonary artery systolic pressure was 44 mmHg with mild pulmonary hypertension. The right atrium was mildly dilated whereas the left atrium was severely dilated with a large mobile mass attached to the wall by a thin stalk, however, the attachment site was not well visualized.

**Figure 2:**
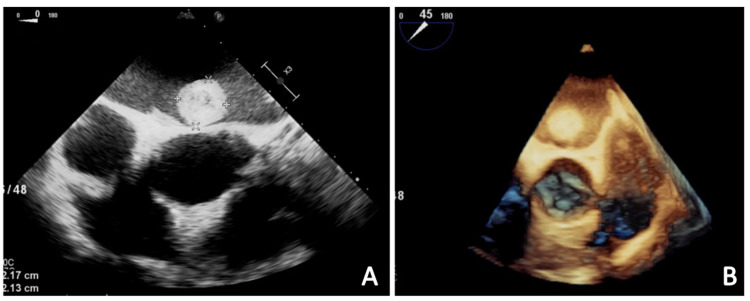
Mid-esophageal view from the TEE with color Doppler (A) and three-dimensional mid-esophageal view from the TEE (B). A large round mobile mass measuring 2.27 cm x 2.17 cm is visible in the left atrium in both images; the mass is hyperechoic and vacuolated in appearance. TEE: transesophageal echocardiography

Transesophageal echocardiography (TEE) was performed which showed a large vacuolated hyperechoic mass measuring 2.27 cm x 2.17 cm in the left atrium, as well as a left atrial appendage (LAA) thrombus (Figure 2 and Videos 1, 2). Given the findings, the patient was started on full-dose anticoagulation with Lovenox (1.5 mg/kg/day) for his LAA thrombus after the third CT scan of the head showed no changes in his hemorrhagic stroke. Cardiothoracic surgery was consulted and he was not deemed to be a candidate for open-heart surgery due to acute hemorrhagic and metastatic neoplasm.

**Video 1:** A large round mobile mass that is hyperechoic and vacuolated in appearance is visible in the left atrium (modality: TEE with color Doppler; view: mid-esophageal; window: short-axis aortic valve). TEE: transesophageal echocardiography

**Video 2:** A large round mobile mass measuring 2.27 cm x 2.17 cm is visible in the left atrium (modality: three-dimensional TEE; view: mid-esophageal; window: short-axis aortic valve). TEE: transesophageal echocardiography

**Figure 3:**
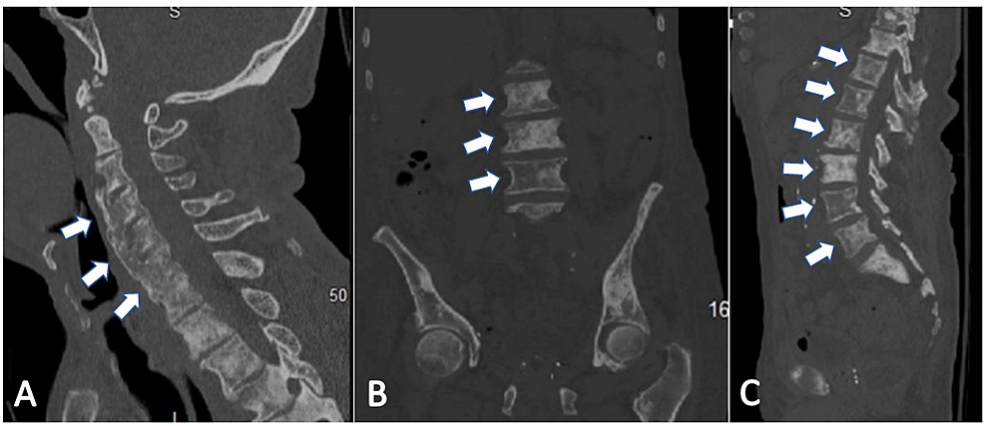
Sagittal and coronal views of the patient's CT scans. (A) Sagittal view of the patient’s CT cervical spine shows diffuse heterogeneous and sclerotic appearance of the visualized vertebral bodies (white arrows) consistent with metastasis from prostate cancer. (B and C) Coronal and sagittal views of the patient’s abdominal CT, respectively, show multiple ill-defined sclerotic lesions throughout the visualized skeleton (white arrows) compatible with sclerotic metastatic disease in the setting of prostate cancer.

In regards to the rest of his medical management, the patient was found to be septic in the setting of positive urinalysis and was treated with ceftriaxone. He received dextrose for hypoglycemia. A percutaneous endoscopic gastrostomy (PEG) tube was placed. His hospital course was complicated by several episodes of hematochezia secondary to rectal tube insertion which caused a drop in his hemoglobin levels. Lovenox and aspirin were held and the patient underwent transfusion with two units of packed red blood cells. Gastroenterology was consulted who recommended an endoscopy, however, the patient’s daughter declined. The patient was started on intravenous pantoprazole. Hematochezia eventually resolved. He was also found to have *Clostridioides difficile* colitis for which he received oral vancomycin. The patient’s acute kidney injury resolved after intravenous fluid administration. Hematology was consulted given his stage IV prostate cancer. CT scans of the spine were remarkable for diffuse heterogeneous and sclerotic appearance of the vertebral bodies and ribs consistent with metastasis from prostate cancer (Figure 3). No acute oncological intervention was recommended during the admission given the patient's poor clinical status and poor prognosis. Palliative care was consulted for goals of care discussion, the patient was made do not resuscitate/do not intubate (DNR/DNI) as per family’s wishes, planned for comfort care and discharge to hospice.

## Discussion

The differential diagnosis of a left atrial mass includes benign or malignant (primary vs. metastatic) tumors, thrombi, or valvular vegetations. The echocardiographic study in our patient showed a vacuolated hyperechoic mass in the left atrium tethered by a septal stalk. This finding supports the differential of a benign cardiac mass, e.g., myxoma, papillary fibroelastoma, or lipoma. Myxomas are the most common PCT and approximately 75% originate in the left atrium [9]. They may appear with finger-like projections or with a smooth surface and could present in homogeneous areas of hyperechogenicity due to calcification [10]. Cardiac sarcomas are the predominant subtype of malignant PCTs, and although they are a rare possibility, can also be found in the left atrium and remain as one of our differentials [2]. It is also possible that the mass is a cardiac thrombus given the hypercoagulable state present in this patient due to advanced cancer. Additionally, the presence of atrial fibrillation and an already formed LAA thrombus in this patient provides further support for the mass being thrombotic in nature. Although exceedingly rare, the prostatic origin of the mass cannot be ruled out given stage IV prostate cancer with extensive sclerotic metastasis. Malignant melanomas are most likely to metastasize to the heart. Carcinomas originating from the lung, breast, esophagus, thyroid, kidneys, and liver are also commonly associated with cardiac involvement [7,8]. However, metastasis from prostate cancer is yet to be reported.

To better distinguish between myxoma vs. thrombi vs. malignant tumor, a TTE with ultrasound enhancing agent (UEA) would have been appropriate in our patient. A myxoma would demonstrate partial perfusion on visual inspection and quantitatively less perfusion compared to the surrounding myocardium. A highly vascularized malignant tumor would present with greater UEA enhancement than the adjacent myocardium, and thrombi would show complete absence of perfusion due to their avascular nature [11]. A multi-modal imaging approach with cardiac magnetic resonance (CMR) and cardiac computed tomography (CCT) in addition to echocardiographic evaluation would have provided a more in-depth understanding of the mass’ characteristics, however, our institution did not have CMR and CCT at our disposal [12,13].

Histopathological characterization following biopsy of resected cardiac mass would have provided a definitive diagnosis. Myxomas are composed of spindle cells, myxoid matrix, vascular structure with often hemorrhagic and calcified areas. A thrombus would present with fibrin network, blood cells, fibroblasts, and granulation tissue depending on its age [14]. In metastasis from prostate cancer, one would expect to see microacinar, tubulopapillary, or carcinoid-like histologic patterns [15]. Complete resection of the mass with subsequent biopsy is indicated; however, our patient is not an appropriate surgical candidate.

## Conclusions

In summary, a proper differential diagnosis is crucial in the field of cardiac masses in order to start the appropriate management. The top differentials for our case include PCT, thrombi, and metastasis. Although a full diagnostic workup was not completed due to limitations in diagnostic tests available, metastasis to the heart could not be excluded due to the high staging and extensive sclerotic involvement of his malignancy. We recommend UEA echocardiography, CCT, CMR, and resection with subsequent biopsy for further diagnostic evaluation.
